# Patch-Based Principal Component Analysis for Face Recognition

**DOI:** 10.1155/2017/5317850

**Published:** 2017-07-11

**Authors:** Tai-Xiang Jiang, Ting-Zhu Huang, Xi-Le Zhao, Tian-Hui Ma

**Affiliations:** School of Mathematical Sciences, University of Electronic Science and Technology of China, Chengdu 610054, China

## Abstract

We have proposed a patch-based principal component analysis (PCA) method to deal with face recognition. Many PCA-based methods for face recognition utilize the correlation between pixels, columns, or rows. But the local spatial information is not utilized or not fully utilized in these methods. We believe that patches are more meaningful basic units for face recognition than pixels, columns, or rows, since faces are discerned by patches containing eyes and noses. To calculate the correlation between patches, face images are divided into patches and then these patches are converted to column vectors which would be combined into a new “image matrix.” By replacing the images with the new “image matrix” in the two-dimensional PCA framework, we directly calculate the correlation of the divided patches by computing the total scatter. By optimizing the total scatter of the projected samples, we obtain the projection matrix for feature extraction. Finally, we use the nearest neighbor classifier. Extensive experiments on the ORL and FERET face database are reported to illustrate the performance of the patch-based PCA. Our method promotes the accuracy compared to one-dimensional PCA, two-dimensional PCA, and two-directional two-dimensional PCA.

## 1. Introduction

The principal component analysis, one of the most popular* multivariate statistical techniques* [1], has been widely used in the areas of pattern recognition and signal processing [2]. It is a statistical method under the broad title of * factor analysis* [3]. The modern instantiation PCA was formalized by Hotelling [1, 4] who also coined the term* principal component*, but in fact we can trace its origin back to [5] or even Cauchy [6]. PCA analyzes the observed data which is usually described by several dependent and intercorrelated variables. Its goal is to extract the important information from the data and to express this information as a set of new orthogonal variables called principal components.

There are numerous PCA-based methods for face recognition, from one-dimensional PCA [7] to two-directional two-dimensional PCA known as (2D)^2^PCA [8]. All these methods rely on two points. Firstly, the pattern of similarity of the observations and the variables can be represented as points in maps by PCA [2, 9, 10]. Secondly, the similarity of face images can be in some sense “calculated” by evaluating the distance of these points.

The main idea of one-dimensional PCA method for face recognition is* eigenspace projection*. A projection matrix is obtained by maximum the image covariance, which shows the correlation between pixels in each training data (or say labeled face image). The next step is projecting the 1D vectors (previously constructed from 2D images) into the feature space [11]. In addition, the eigenvectors corresponding to large eigenvalues (or say the principle components), which would resemble a human face after transforming into matrix of the same size of the original face image, are called* eigenface*. Then the nearest neighbor (NN) classifier is adopted by computing the distance in the eigenspace to verify the identity of unlabeled face images. For instance, we would be sure that the face belongs to the 1st individual, if an unlabeled face image is nearest to one of the 1st individual's labeled face images in the eigenspace. However transforming 2D images into 1D vectors always leads to a very high-dimensional space, in which the calculating of the covariance matrix, which shows the correlation of pixels, is difficult. The size of the covariance matrix achieves 10000 × 10000, if the size of face images is 100 × 100. Hence, it would consume a lot of time to evaluate the eigenvectors of a such large size covariance matrix.

Two-dimensional principal component analysis (2DPCA) [12], as opposed to eigenface, projects face images into a subfeature space directly without image-to-vector conversion. This direct projection not only enables the preservation of partial image spatial information but also reduces computational burden [13]. The so-called image covariance matrix of 2DCPA, which is constructed directly using the original face image matrixes, is much smaller than the covariance matrix of eigenface method. In 2DPCA, the image covariance (scatter) matrix, which is somehow the same as the covariance matrix in the eigenface, shows the correlation of each column of each image. Motivated by 2DPCA, (2D)^2^PCA [8] calculate the correlation from two directions of both of the columns and rows. 2DPCA and (2D)^2^PCA have achieved good results in face recognition. However these methods fail to fully explore the local spatial information.

In order to further explore the local spatial information, let us take a look at the track of existing methods. Eigenface method only calculates the correlation of pixels, while the 2DPCA only calculates the correlation of columns. And (2D)^2^PCA calculates the correlation of both columns and rows in the same time. Accuracy is promoted from one-dimensional PCA to (2D)^2^PCA, when the basic unit is changing from pixels to both columns and rows. Then what is the best basic unit if this evolution continues? We believe that patch is the most meaningful basic unit for these linear classification methods (e.g., people is discerned by eyes and nose). The local spatial information of eyes and nose is contained in the patches. So it is more intuitive to consider the correlation of different patches. From another aspect, patch is successfully used in the field of image processing recently, not only face recognition [14–16] but also image denoising [17–19], image superresolution [20, 21], and image decomposition (cartoon-texture [22, 23] or illumination-reflectance [24] and further retinex image enhancement [25]). Patch is becoming a basic tool in these above-mentioned literatures. Motivated by our idea that the patch is the most meaningful basic unit for these linear classification methods and the widely successful application of patch, we intend to calculate the correlation of the patches in the computation of our PCA.

For the purpose of calculating the correlation of the patches, we simply add patch preprocessing before the frame work of 2DPCA. That is, we first divide the face images into patches and then we convert these patches into columns. The columns, in 2DPCA frame work, are substituted by our patch-unfold-columns, so the correlation between columns in the 2DPCA becomes the correlation between patches after calculating the image covariance (scatter) matrix. Then the orthonormal eigenvectors of the image covariance (scatter) matrix can be the optimal projection axes which are used for feature extraction. The optimal projection axes are used to form a matrix, which is called the feature matrix or feature image of the training images [12]. The test images are projected on this projection matrix and then classified by finding out the nearest neighbor of the projections of the test images. We call this method* patch-based principal component analysis* (PPCA). As a result, the main contribution of the proposed method is that the most meaningful basic unit patch is incorporated in the frame work of 2DPCA, so that the correlation between the most meaningful basic units is utilized to promote the accuracy rate. This is confirmed our experiments. Besides, the proposed method can be easily implemented.

In fact, we can choose the support vector machine (SVM) as classifier and this may improve the accuracy rate. But SVM is not necessary for the comparison among our method and eigenface method, 2DPCA, and (2D)^2^PCA. In another aspect, we know that PCA is one of global techniques [26], so that it is difficult to utilize both the local spatial correlation between pixels in each patch and the nonlocal spatial correlation between patches as [17]. But we consider that the global computation could somehow compensate the utilization of the nonlocal spatial correlation between patches.

It is noteworthy that there has been great progress of face recognition nowadays. It is very hard for an improved version of an old method to challenge the recent deep learning [27, 28] based methods. Please refer to [29] for a more extensive overview on face recognition. However the improvement of an old method is still meaningful, since that many old methods are being widely employed, e.g., the alternating direction method of multipliers (ADMM) [30–35] and block coordinate decent (BCD) algorithm [36]. Meanwhile, what we focus on is the improvement of the PCA-based classification method. Moreover, the experimental results in Section 3 have indeed validated that our method outperforms other PCA-based methods.

The outline of this paper is given as follows. In Section 2, we present our PPCA method for face recognition. In Section 3, experimental results are reported to demonstrate the performance of the proposed method. Finally, some conclusions are drawn in Section 4.

## 2. Patch-Based Principal Component Analysis

In 2DPCA, an image matrix **A**_image_ of size *n*_1_ × *n*_2_ is directly projected on *n*_2_-dimensional unitary column vectors: **Y** = **A**_image_**X**. By maximizing the total scatter *J*(**X**) = tr{*E*[(**Y** − *E ***Y**)(**Y** − *E ***Y**)^*T*^]} = tr{**X**^*T*^*E*[(**A** − *E ***A**)^*T*^(**A** − *E ***A**)]**X**}, we obtain the projection matrix. Then the following steps are feature extraction and classification. Our PPCA just adds a patch preprocessing prior to this frame work above. Then, same as 2DPCA, we calculate the image covariance matrix and optimal projection axes for feature extraction and classification.

### 2.1. Patch Preprocessor

Suppose that we have *m* training facial images. For the *j*-th training sample, we divide the image of size *n*_1_ × *n*_2_ into *N* patches of size *p* × *q*  (1 ≤ *p* ≤ *n*_1_, 1 ≤ *q* ≤ *n*_2_). If *n*_1_ (or *n*_2_) is not divisible by *p* (or *q*), we would add overlap *θ*_1_ (or *θ*_2_), so that (*n*_1_ − *θ*_1_)/(*p* − *θ*_1_) (or (*n*_2_ − *θ*_2_)/(*q* − *θ*_2_)) would always be integer no matter the choice of *p* (or *q*). Generally, for the sake of reducing computational burden, we choose the smallest one of the overlaps for each selected *p* (or *q*). Then we can get the number of patches of every face image:

(1) $$\begin{array}{c} N\left(\;p,q\;\right)=\frac{n_{1}\times n_{2}}{p\times q}, \end{array}$$

or with the overlap (*θ*_1_, *θ*_2_)

(2) $$\begin{array}{c} N\left(\;p,q,\theta_{1},\theta_{2}\;\right)=\frac{\left(n_{1}-\theta_{1}\right)\times \left(n_{2}-\theta_{2}\right)}{\left(p-\theta_{1}\right)\times \left(q-\theta_{2}\right)}. \end{array}$$

Then we convert each patch into a column vector of size *M*( = *p* × *q*):

(3) $$\begin{array}{c} a_{i}=\left(\begin{array}{c} p_{1}\\ p_{2}\\ \vdots \\ p_{M} \end{array}\right),\;\left(i=1,2,\ldots ,N\right), \end{array}$$

More details about the patch-to-vector conversion are given in the Section 3. Then let *A*_*j*_ represent all of the reshaped vectors of the *j*-th training facial image

(4) $$\begin{array}{c} A_{j}=\left[\;a_{1},a_{2},\ldots ,a_{N}\;\right], \end{array}$$

where the size of **A**_*j*_ is *M* × *N*, and *j* = 1,2,…, *m*.

It should be noted that we adopt the 2D-PCA framework rather than (2D)^2^PCA. As mentioned before, (2D)^2^PCA takes both the correlations of columns and rows into consideration, while the 2DPCA method concentrates on the correlations between column vectors. Meanwhile our patch preprocessing convert patches into vectors. Therein, it is reasonable to adopt the 2D-PCA framework rather than (2D)^2^PCA.

### 2.2. Total Scatter

Let **X** ∈ *ℝ*^*N*×*d*^ be a matrix with orthonormal columns, *N* > *d*. Then we project matrix **A** of size *M* × *N* onto **X** by the following linear transformation [37, 38]:

(5) $$\begin{array}{c} Y=AX. \end{array}$$

**Y** is an *M*-dimensional projected vector (i.e., the projected feature vector [12]) of matrix **A**. Same as 2DPCA, we use the total scatter of the projected samples to measure the discriminatory power of the projection matrix **X**:

(6) JXtrEY−EYY−EYT=trEAX−EAXAX−EAXT=trXTEA−EATA−EAX.

Let us define

(7) $$\begin{array}{c} G≔E\left[\;{\left(\;A-EA\;\right)}^{T}\left(\;A-EA\;\right)\;\right], \end{array}$$

which is called the image covariance (scatter matrix). The average matrix of all the *L* preprocessed images is

(8) $$\begin{array}{c} \bar{A}=\frac{1}{L}\overset{L}{\underset{j=1}{\sum }}A_{j}. \end{array}$$

Then **G** can be evaluated by

(9) $$\begin{array}{c} G=\frac{1}{L}\overset{L}{\underset{j=1}{\sum }}{\left(\;A_{j}-\bar{A}\;\right)}^{T}\left(\;A_{j}-\bar{A}\;\right). \end{array}$$

It is easy to verify that **G** is a semipositive matrix. We can evaluate **G** directly using the *L* training samples. The total scatter of the projected samples can be expressed by

(10) $$\begin{array}{c} J\left(\;X\;\right)=X^{T}GX, \end{array}$$

where **X** is a unitary column vector. This is called* generalized total scatter criterion* [12]. The unitary vector **X** is called the optimal projection axis that maximizes the criterion.

### 2.3. Optimization

It has been proved that the optimal projection axis **X**_opt_, which maximizes the total scatter of the projected samples, is the eigenvectors of **G** corresponding to the largest eigenvalues [38]. In general, we choose the orthonormal eigenvectors **X**_1_,…, **X**_*d*_ of **G** corresponding to the first *d* largest eigenvalues. They are equivalent to

(11) X1,…,Xd=arg maxX  JX, XiTXj=0,i≠j,  i,j=1,…,d.

The first eigenvector is required to have the largest possible variance (i.e., this component will “explain” or “extract” the largest part of the pattern information of the preprocessed face images [1]). We can simply control the value of *d* by a threshold *θ* as follows [8]:

(12) $$\begin{array}{c} \frac{\sum_{i=1}^{d}\lambda_{i}}{\sum_{i=1}^{N}\lambda_{i}}\geq \theta , \end{array}$$

where *λ*_*i*_ (*i* = 1,2,…, *N*) are the first *N* largest eigenvalues. We can determine *d* by presetting *θ* or even referring to the results from different face database.

### 2.4. Feature Extraction and Classification

For each* patch-preprocessed* facial image in training set **A**_*j*_, let

(13) $$\begin{array}{c} Y_{j}=A_{j}X, \end{array}$$

where **X** = [**X**_1_,…, **X**_*d*_] of size *N* × *d* is the projection matrix. We call **Y**_*j*_ = [**Y**_*j*_^(1)^,…, **Y**_*j*_^(*d*)^] of size *M* × *d* the* patch-based feature matrix* and **Y**_*j*_^(*i*)^ (*i* = 1,…, *d*) the* patch-based principal components (vectors*) of the *j*-th sample image.

After patch preprocessing and 2DPCA projection, facial images in the training set have been transformed into the* patch-based feature matrixes*. We use the nearest neighbor (NN) classifier [39] for classification. We define the distance between two arbitrary patch-based feature matrixes by

(14) $$\begin{array}{c} d\left(\;Y_{i},Y_{j}\;\right)=\overset{d}{\underset{k=1}{\sum }}{\left\|\;Y_{i}^{\left(k\right)}-Y_{j}^{\left(k\right)}\;\right\|}_{2}, \end{array}$$

where ‖·‖_2_ denotes the Euclidean distance.

We have *L* training facial images, each of which is assigned a given identity. Given a test facial image, we first do a patch preprocessing and obtain a preprocessed matrix **A**_test_. Then we project **A**_test_ onto **X** and obtain **Y**_test_. If

(15) dYtest,Yl=minj dYtest,Yj≤ω,

where *ω* is a preset thresholding, the test image results to the same kind of **Y**_*l*_, that is, the test facial image and the *l*-th training image, belongs to the same person. Otherwise, if min_*j*_⁡ *d*(**Y**_test_, **Y**_*j*_) ≥ *ω*, the test sample does not belong to any identity in this training data.

## 3. Experimental Results

In this section, the performance among our proposed PPCA and the eigenface method (or say the 1DPCA method), the 2DPCA method, and the (2D)^2^PCA method is evaluated on two well-known face image databases (ORL and FERET). To our point of view, experiments on constrained face databases are sufficient to validate the superiority of the proposed method among these methods. Thus, unconstrained face databases, for example, LFW database, are not taken into consideration.

First, the recognition accuracies of these four methods are compared with the experimental strategy that use half of the images in the database for training. After that, more experimental results show the influence from reordering and the size of patches. All experiments are performed using Matlab (R2014a) on a desktop with 3.40 GHz Intel core i7-2600 CPU and 12 GB RAM equipped with Windows 7 OS. If not specified, the preset threshold *θ*, which controls the number of projection vectors, is set to 0.90 in the latter experiments. That is, we extract 90 percentage energy of the whole training images.

### 3.1. Recognition Accuracy Results on the FERET Database

**Figure 1:**
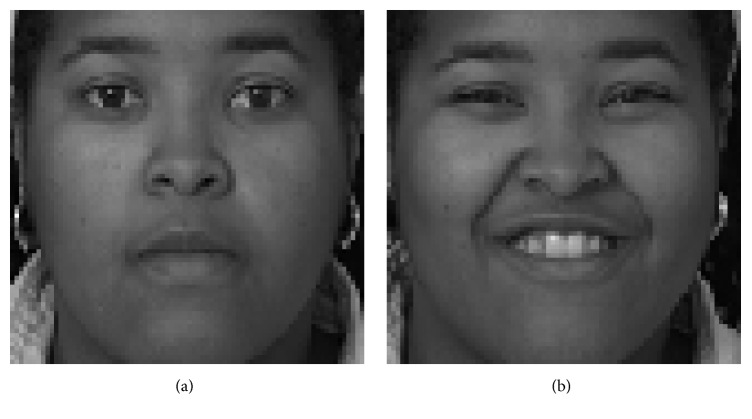
Two different images of one individual in the partial FERET database. (a) belongs to the subset** fa**, while (b) belongs to the subset** fb**.

The FERET database [40, 41] is a standard dataset used for facial recognition system evaluation. The Face Recognition Technology (FERET) program is managed by the Defense Advanced Research Projects Agency (DARPA) and the National Institute of Standards and Technology (NIST). Until 2003, there are 2,413 facial images representing 856 individuals in the FERET database. The performance of the above 4 methods are tested on the partial FERET face database, which contains 400 images (with the cropped size 80 × 80) from 200 individuals, each providing 2 different images. The so-called** fa** subset, which contains 100 images, is used as training data, while the so-called** fb** subset, containing remaining 100 images, is used as testing data.  Figure 1 shows 2 images of one individual in the ORL database.

From Table 1, we can see that the PPCA method achieves the highest accuracy on the FERET database. To get the highest accuracy, parameter *θ* is set referring to the results. The recognition accuracy is improved from 84.0 percentage of 2DPCA and 83.0 percentage of (2D)^2^PCA to 86 percentage. It means that the PPCA method recognized 2 more images than 2DPCA and 3 more images than (2D)^2^PCA on the FERET database. We remarked here that images of cropped size 60 × 60 were used in [8] and 83%, 84.5%, and 85% accuracy rates were got, respectively, by 1DPCA, 2DPCA, and (2D)^2^PCA.

### 3.2. Recognition Accuracy Results on the ORL Database

**Figure 2:**
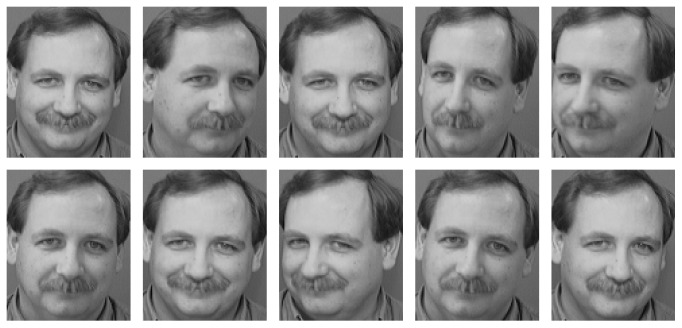
10 different images of one individual in the ORL database. First 5 images are used as training data while the other 5 images are used as testing data.

The ORL database contains images from 40 individuals, each providing 10 different images with the size 112 × 92 (http://rduin.nl/prhtml/prdatafiles/orl.html).  Figure 2 gives 10 images of 1 individual in the ORL database. As previously mentioned, the first five images of each individual are used as training data, and the remaining five images are used as testing data.

Table 2 gives the results on the comparisons of the four methods on recognition accuracy. Both 2DPCA and (2D)^2^PCA reach 90.5% accuracy, which is higher than eigenface method. Our method achieves the highest accuracy on this database. The recognition accuracy is improved from 90.5 percentage to 91.0 percentage with four different sizes of the patch. That is, the PPCA method could recognize 1 more face image than 2DPCA and (2D)^2^PCA on the ORL database. The CPU time of the PPCA method is not desirable but less serious in its consequences.

### 3.3. Influence of Reordering the Patch

The patch-to-vector conversion has a significant impact on the performance of our method. Our initial patch preprocessor converts a patch into a column vector by directly concatenating small columns in the patch. This indeed increased the recognition accuracy that our method achieves 91.0% recognition accuracy with four different sizes of patch. However this improvement does not satisfy us. Employing the idea of clustering, we convert a patch into a column vector by reordering pixels by values for the sake of placing the approximative values together. The concatenating strategy is compared with reordering strategy by contrasting the results of recognition accuracy and CPU time on five different sizes of patch in Table 3.

**Figure 3:**
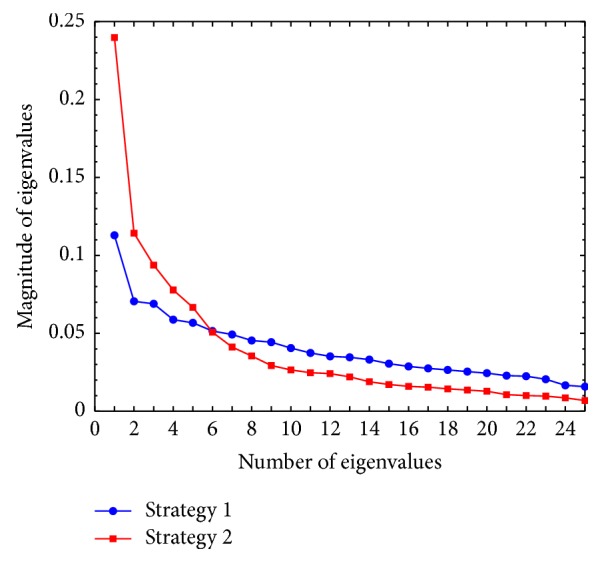
The plot of the magnitude of the eigenvalues of in decreasing order. Strategy  1 represents the “concatenating” strategy, while strategy  2 represents “reordering” strategy.

Table 3 shows that the reordering strategy achieves better performances on recognition accuracy than concatenating strategy. Although reordering strategy implies an additional step of ranking the values in order, its CPU time is not always more than concatenating strategy. We further analyze the eigenvalues of the image covariance (scatter matrix) **G**, which is defined in (9). The patch size 24 × 20 is selected and the comparison is conducted on the ORL database. Here, the size of image covariance matrix **G** was 25 × 25, so it was very easy to calculate its eigenvalues. In Figure 3, the magnitude (eigenvalues divided by the sum of eigenvalues) of the eigenvalues by these two strategy is plotted in decreasing order.

As depicted in Figure 3, the magnitude of the eigenvalues with strategy  2 decreases faster than that with strategy  1. That is, the first small number of eigenvalues by reordering strategy is larger than the same number of eigenvalues by concatenating strategy. This implies that the energy of a patch-preprocessed facial image is concentrated on its first small number of component vectors. Therefore, it is reasonable to use these component vectors for recognition purposes [12]. In addition, the more concentrated the energy on the first small number of eigenvalues is, the smaller the value of *d* in (11) would be. The smaller value of *d* brings less computation complexity and less CPU time, which is exactly consistent with the results of CPU time in Table 3.

We remarked here that though reordering strategy achieves higher accuracy on the ORL database, we have to admit that it may not be stable. Reordering strategy does not achieve higher accuracy than concatenating strategy on the FERET database. This potential instability may be attributed to the patch-to-vector procedure, which might more or less lose the structural information. Hence, in our further work, we will attempt to find better ways to preserve more local spatial structural information rather than better strategies for patch-to-vector conversion.

### 3.4. Influence of the Patch Size

The PPCA method can somehow be considered as a generalization of 2DPCA method. 2DPCA method is a particular case of the PPCA method when the patch size is *n*_1_ × 1. When the size of patch is 1 × 1 or *n*_1_ × *n*_2_, the PPCA method resembles one-dimensional PCA.  Table 4 illustrates that the choice of patch size affects the performance of our method both on the recognition accuracy and on CPU time. A bad choice of patch size might generate a negative result. We would better not choose patches with too large or too small sizes. Therefore, it is important but not easy to choose a patch size with high recognition accuracy. Besides, the computation complexity is so large when the patches are highly overlapped (e.g., patch size 20 × 19 with overlaps 0 and 18). That is, our method would take much more time than 1DPCA, 2DPCA,and (2D)^2^PCA, if the patches are highly overlapped. Hence, it is better to choose patches of moderate sizes with small overlaps.

With the further analysis of the results, we find that there is a difference among the results who are identified of different patches. Our early experiment shows that 2DPCA and (2D)^2^PCA both get the same results of identified people. We can conclude that their capability of identification is same. Thus, the results of our method are compared with results of 2DPCA. The results, with respect to 2DPCA, on the ORL database from two strategies are shown, respectively, in Tables 5 and 6. The item named “number of more identified images” refers to the number of facial images in the testing set which our method correctly recognizes but 2DPCA fails to identify. The item named “number of images failed to be identified” refers to the number of facial images in the testing set which our method does not identify but 2DPCA recognizes.

From Table 4, we can find that the 195th face image in the test set is not recognized by our method of patch size 2 × 24, while the 42nd and 133rd are recognized in contrast with results given by 2DPCA and (2D)^2^PCA. And the 200th face image in the test set is recognized with the patch size of 4 × 17. The 69th and 133rd face images are recognized and the 198th failed to be identified with the patch size of 31 × 23. The 42nd and 200th face images are recognized and the 152nd is not identified with the patch size of 24 × 11.  Table 5 gives the comparison of our method, with different size of patches and “sorting” strategy. For the sake of simplicity, the details would no longer be listed.

We can see from Table 4 that different sizes of patches bring different identifying results though they achieve the same accuracy. As observed from Table 5, the PPCA method with similar size of patches performs approximately the same. For instance, our method with patch sizes 38 × 6 and 38 × 6 recognizes the 69th image in the ORL database, while patch sizes 24 × 11, 24 × 11, and 24 × 11 do not contribute to the recognition of the 69th image.

These differences in the results between our PPCA and 2DPCA (and (2D)^2^PCA), differences among our method from different size of patches, and similarities in results from similar size of patches reveal that the capability of extracting different features would differ on account of the choice of patch size. This indeed validates our belief that* “patch is the meaningful basic unit for classification (e.g., people is discerned by eye and nose),”* with being aware that eye or nose and so forth are of different sizes.

## 4. Conclusions

We have presented a patch-based PCA method to deal with face recognition. By simply doing a patch preprocessing, before the computation of projection matrix of 2DPCA, we can directly calculate the correlation of the patches instead of the rows or columns of face images. Comparisons of recognition accuracy are made with the 1DPCA [7], 2DPCA [12], and (2D)^2^PCA [8] methods on the ORL face database and the FERET database. Numerical experiments are represented to illustrate that the use of patch promotes the accuracy compared to former 1DPCA, 2DPCA, and (2D)^2^PCA. Meanwhile, the results demonstrate our belief that patch is the most meaningful basic unit for classification.

## Figures and Tables

**Table 1:** Comparison of the four methods on recognition accuracy on the partial FERET database.

Method	Accuracy (%)	Patch size (pixel)	*θ*_1_	*θ*_2_	Time (s)	Threshold *θ*
1DPCA	79.0	—	—	—	1.454	0.99
2DPCA	84.0	—	—	—	1.734	0.80
(2D)^2^PCA	83.0	—	—	—	2.104	0.90
PPCA	**86**	20 × 19	0	18	26.658	0.92

**Table 2:** Comparisons of the four methods on recognition accuracy on the ORL database.

Method	Accuracy (%)	Patch size (pixel)	*θ*_1_	*θ*_2_	Time (s)
1DPCA	88.0	—	—	—	2.673
2DPCA	90.5	—	—	—	5.026
(2D)^2^PCA	90.5	—	—	—	4.044
PPCA	**91.0**	2 × 24	0	7	7.186
		4 × 17	0	2	6.677
		31 × 23	4	0	8.617
		24 × 11	2	2	7.475

**Table 3:** Comparison of two strategies on recognition accuracy and CPU time on the ORL database.

Patch size	*θ*_1_	*θ*_2_	Concatenating		Reordering	
			Accuracy (%)	Time (s)	Accuracy (%)	Time (s)
24 × 11	2	2	91.0	**8.093**	**92.0**	8.523
24 × 20	2	2	90.5	8.356	**93.0**	**7.924**
24 × 23	2	0	90.0	**7.702**	**93.5**	9.060
38 × 6	1	4	90.0	13.184	**93.0**	**10.873**
38 × 7	1	2	90.0	**7.053**	**93.0**	8.335

**Table 4:** Comparison of different patch sizes on recognition accuracy and CPU time on the ORL database and the FERET database.

ORL database					FERET database				
Patch size	*θ*_1_	*θ*_2_	Accuracy (%)	Time (s)	Patch size	*θ*_1_	*θ*_2_	Accuracy (%)	Time (s)
2 × 2	0	0	87.0	21.553	2 × 2	0	0	75.5	9.175
28 × 8	0	1	87.5	5.584	2 × 19	0	18	85.0	36.974
24 × 11	2	2	91.0	7.475	10 × 9	0	8	82.0	12.490
22 × 23	4	0	89.0	7.835	10 × 18	0	16	84.0	13.793
24 × 23	2	0	90.0	8.356	16 × 13	0	12	83.0	17.326
31 × 20	4	2	90.5	8.832	20 × 19	0	18	86.0	30.658
40 × 40	4	14	86.5	10.076	40 × 40	0	18	79.0	6.855

**Table 5:** Differences (with respect to 2DPCA) in the identifying results with different size of patches and “concatenating” strategy.

Patch size (pixel)	Accuracy (%)	*θ*_1_	*θ*_2_	Number of more identified images	Number of images failed to be identified
2 × 24	91.0	0	7	42, 133	195
4 × 17	91.0	0	2	200	NA
31 × 23	91.0	4	0	60, 133	198
24 × 11	91.0	2	2	42, 200	152

**Table 6:** Differences (with respect to 2DPCA) in the identifying results with different size of patches and “sorting” strategy.

Patch size (pixel)	Accuracy (%)	*θ*_1_	*θ*_2_	Number of more identified images	Number of images failed to be identified
24 × 11	92.0	2	2	25, 50, 53, 98, 176, 180, 196, 200	49, 83, 84, 114, 160
24 × 20	93.0	2	2	25, 50, 53, 98, 176, 180, 196, 200	83, 84, 160
24 × 23	93.5	2	0	25, 42, 50, 53, 98, 176, 180, 196, 200	83, 84, 160
38 × 6	93.0	1	4	25, 50, 53, 69, 98, 176, 180, 196, 200	49, 83, 84, 160
38 × 7	93.0	1	2	25, 42, 50, 53, 69, 98, 176, 180, 196, 200	49, 83, 84, 134, 160
